# Evolution of Cooperation on Stochastic Dynamical Networks

**DOI:** 10.1371/journal.pone.0011187

**Published:** 2010-06-30

**Authors:** Bin Wu, Da Zhou, Feng Fu, Qingjun Luo, Long Wang, Arne Traulsen

**Affiliations:** 1 Center for Systems and Control, State Key Laboratory for Turbulence and Complex Systems, College of Engineering, Peking University, Beijing, China; 2 Emmy-Noether Group for Evolutionary Dynamics, Max-Planck-Institute for Evolutionary Biology, Plön, Germany; 3 School of Mathematical Sciences, Peking University, Beijing, China; 4 Program for Evolutionary Dynamics, Harvard University, Cambridge, Massachusetts, United States of America; 5 School of Electronics Engineering and Computer Science, Peking University, Beijing, China; Indiana University, United States of America

## Abstract

Cooperative behavior that increases the fitness of others at a cost to oneself can be promoted by natural selection only in the presence of an additional mechanism. One such mechanism is based on population structure, which can lead to clustering of cooperating agents. Recently, the focus has turned to complex dynamical population structures such as social networks, where the nodes represent individuals and links represent social relationships. We investigate how the dynamics of a social network can change the level of cooperation in the network. Individuals either update their strategies by imitating their partners or adjust their social ties. For the dynamics of the network structure, a random link is selected and breaks with a probability determined by the adjacent individuals. Once it is broken, a new one is established. This linking dynamics can be conveniently characterized by a Markov chain in the configuration space of an ever-changing network of interacting agents. Our model can be analytically solved provided the dynamics of links proceeds much faster than the dynamics of strategies. This leads to a simple rule for the evolution of cooperation: The more fragile links between cooperating players and non-cooperating players are (or the more robust links between cooperators are), the more likely cooperation prevails. Our approach may pave the way for analytically investigating coevolution of strategy and structure.

## Introduction

Cooperation is ubiquitous in the real world ranging from genes to multicellular organisms [1]–[4]. Most importantly, human society is based upon cooperation. However this cooperative behavior apparently contradicts natural selection [5]: Selfish behavior will be rewarded during competition between individuals, because selfish individuals enjoy the benefits from the cooperation of others, but avoid the associated costs. Therefore, the puzzle how natural selection can lead to cooperation has fascinated evolutionary biologists since Darwin.

Evolutionary game theory is an intuitive and convenient framework to study this puzzle. As a metaphor, the Prisoner's Dilemma (PD) has been widely used to investigate the origin of cooperation. In this game, two players simultaneously decide whether to cooperate (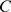) or to defect (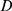). They both receive 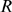 upon mutual cooperation and 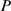 upon mutual defection. A defector exploiting a cooperator receives 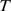, and the exploited cooperator gets 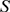. This can be formalized in the form of a payoff matrix,
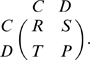
(1)The PD is characterized by the payoff ranking 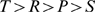. For repeated games, the additional requirement 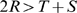 ensures that alternating between strategies is less lucrative than repeated mutual cooperation. In the one shot PD, it is best for a rational individual never to cooperate irrespective of the co-player's decision. Thus, defection is the Nash Equilibrium [6]. However, the two players would be better off if they both cooperated, hence the dilemma. In an evolutionary setting, where payoff determines reproductive fitness, defectors can reproduce faster based on their higher payoff and cooperation diminishes - defection is evolutionary stable [7], [8]. Several mechanisms have been proposed to explain the persistence of cooperative behavior, including kin selection [9], direct [10], [11] and indirect reciprocity [12], [13], group selection [14], [15] as well as the network reciprocity [16]–. Furthermore, the relationship between these mechanisms receives an increasing attention [25]–[28].

Both in animal and human societies, individuals interact with a limited number of individuals. The interactions of individuals are often captured based on the network of contacts. Therefore, there has been an increasing interest in the influence of population structure on the evolution of cooperation.

Nowak and May first studied the PD game on regular lattices [16]. Subsequently, social dilemmas on regular graphs have been investigated [19]–[21], [29]. Many authors have also considered more complex networks, such as scale-free and small-world afterwards [17], [18], [24]. It has been well recognized that network topologies can play a crucial role in the evolution of cooperation, in addition to the payoff matrix and the update mechanism.

The network topology is assumed to be static in the above work. However, social relationships between individuals are not eternal, but are continuously changing in the real world. Therefore, the coevolution of strategy and network receives increasing attention [30]–[47].

Dynamical networks can significantly boost cooperation compared to static networks. On the one hand, cooperation thrives if individuals are able to promptly adjust their social ties, because this allows cooperators to escape from defectors [38]. Similarly, cooperation is more likely to occur if the favored relationships between cooperators (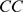 links) tend to be less fragile than adverse social ties (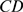 links) [37], [43]. The latter result is consistent with our empirical intuitions and is widely observed in the real world. However, most of the works on this issue are investigated only by numerical methods and not by analytical approaches. This is mainly because it is difficult to describe the coevolution of strategy and structure of a network analytically.

Pacheco *et al.* approximate their linking dynamics by ordinary differential equations [39]–[41], [43]. They found that fast linking dynamics leads to a transformation of the payoff matrix, such that e.g. cooperation in a Prisoner's Dilemma can be stabilized. This approach does not keep the total number of links constant. Moreover, the analytical approach does not take stochastic effects into account.

Here, we consider a linking dynamics described by a discrete stochastic model. The evolution of links can be described as a Markov chain, which is the starting point for our analytical considerations. We specify the conditions required for the payoff matrix to make cooperation stable. A simple rule is obtained when the linking dynamics proceeds sufficiently fast, which reveals quantitatively how the link breaking probabilities have to be chosen such that cooperation may gain a foothold. Furthermore, we show how our stochastic linking dynamics also results in a transformation of the payoff matrix as in [39].

## Analysis

We consider the coevolution of strategy and structure in the PD game. Each player's strategy 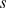 can either be cooperation (C) or defection (D), denoted by 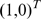 and 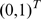, respectively. Initially, the whole population of size 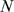 are situated on vertices of a regular graph with degree 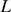, where nodes indicate individuals while edges denote the pairwise partnerships between individuals. We consider the case where the total number of agents 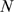 is much larger than the average degree 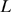. The payoff of each individual is obtained by playing the PD game with all of its immediate neighbors:
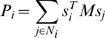
(2)where 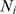 represents the neighborhood set of player 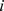 and 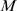 is the payoff matrix. Instead of the general matrix of the Prisoner's Dilemma Eq. (1) with four parameters, we consider a simpler payoff matrix,
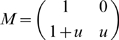
(3)where the parameter 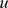, measuring how profitable unilateral defection is, ranges from zero to one. Note that this payoff matrix recovers the payoff ranking described above, 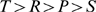.

We emphasize that Eq.(3) describes a special case of general PD games, but it is widely used in biology and sociology [1].

In each time step, an agent has the opportunity to change its strategy with probability 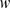. With probability 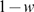, a link in the network can be changed. For 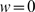, no strategy update takes place, hence the cooperation level stays unchanged and only the dynamical organization of cooperators and defectors can be observed [45]. For 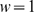, this model degenerates to a PD game on a static regular graph, which has been studied in great detail [16], [19], [22], [29], [48].

**Figure 1:**
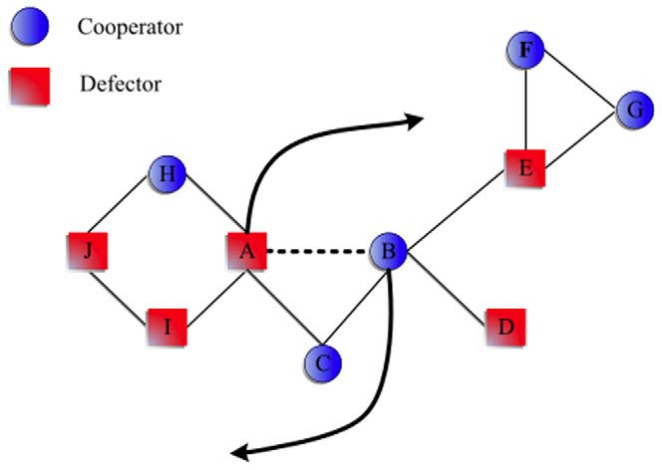
Linking dynamics. If the dashed link is selected in the topological evolution, it will be broken off with probability 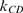. If the dashed link is broken, then either A or B is selected to establish a new link. If A is chosen, then he switches to a random individual of the population who is not his current neighbors (B, D, E, F, G or J). Otherwise, B is chosen, then he also switches to a random individual of the population who is not his current neighbors (A, F, G, H, I or J).

Let us first consider the dynamics of links (which occurs with probability 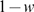). In each rewiring step, a link 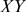 is selected from the network at random (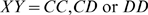). The link remains intact with probability 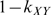. With probability 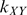, the link is broken. In this case, one of the two adjacent players is picked at random and switches to a random player who is not its immediate neighbor in the population (see Fig. (1)). In this way, link 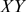 is broken and a new link 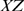 or 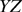 is introduced.

We consider the case of 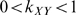. In this case, linking dynamics does not assume rationality of agents: Adverse 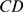 links may be kept and advantageous 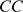 links may sometimes be broken.

In contrast with previous analytical work focusing on a dynamical number of links [39], here the total number of links 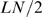 is constant in the evolution process as in [33], [38]. This constraint can imply a limited resource and avoids that all individuals are linked to all others (for generic parameter choices).

In the beginning, each link is assigned a name 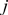, where 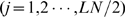. In each time step, we choose a link 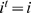 at random, where the superscript denotes the time. If the selected link 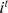 does not break, we have 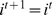. If the link breaks, a new social tie is introduced, denoted as 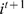. We denote the type of link 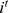 by 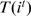, where 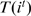 can be 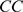, 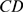 or 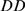. Herein, we investigate how 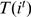 changes with time 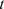.

The dynamics of 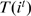 can be captured by a Markov chain with transition matrix 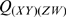, which is the probability that an 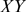 link transforms to a 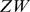 link in one time step. According to the linking dynamics, the probability of moving between 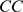 and 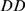 is zero. So, we only have to calculate 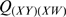 or 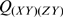.

For instance, 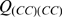 is the probability that 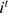 of type 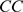 transforms to 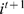 of type 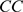. This occurs in the following cases:

1. When 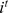 is not selected in the linking dynamics (with probability 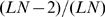);
2. When 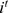 is selected (with probability 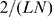), this happens either when the original 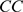 link is not broken off (with probability 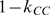) or when the selected player of the original link switches to another cooperator provided the link is broken (with probability 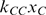 where 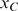 is the frequency of cooperators). Hence,
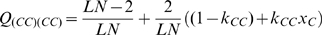
(4)Similar considerations for other links lead to the transition probability matrix
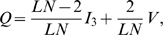
(5)where 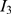 is the identity matrix and the matrix 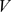 is given by
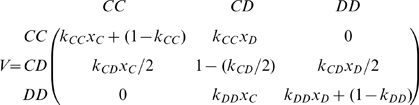
(6)and 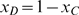 is the frequency of defectors. We emphasize that the transition matrix is only an approximation, because it does not exclude the case that a player establishes a second link with one of its immediate neighbors. However, the approximation is very good when the degree of all links is much smaller than the population size.

Note that this Markov chain is irreducible and aperiodic when 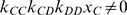, hence there exists a unique stationary distribution 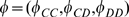 determined by equation 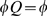 [49]. We find that
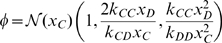
(7)where 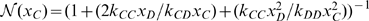 is a normalization factor. Here, 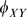 represents the probability that a link 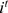 is of type 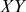 in the stationary regime. Therefore, the average number of 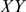 links 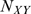 is given by:
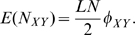
(8)Thus, 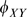 also represents the average fraction of 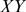 links in the whole population in the stationary regime of the linking dynamics.

Let us now consider the dynamics of strategies (which occurs with probability 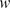). A player 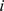 with strategy 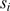 is selected at random, subsequently player 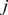 with strategy 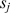 is randomly selected among 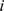
's current neighbors. Player 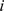 compares the payoff with that of player 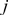 and takes strategy 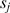 with probability [48], [50]

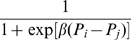
(9)where 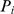 and 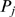 are the accumulated payoffs for 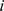 and 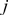, respectively. The parameter 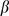 denotes the intensity of selection. For 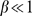, selection is weak and strategy changes are almost random. For 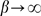, selection is strong and strategies of more successful agents are always adopted, whereas less successful agents are never imitated. In large, well mixed populations the dynamics can be approximated by [51]

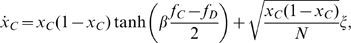
(10)where 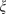 is the Gaussian white noise with variance 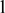, 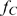 and 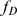 denote the average fitness of a cooperator and a defector, respectively. For large population size 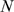, the stochastic term vanishes [51] and we obtain
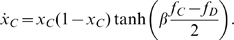
(11)Note that this equation has the same equilibrium properties as the usual replicator dynamics [8]

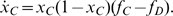
(12)If 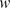 is sufficiently small, the structure of the system is close to the stationary state when strategies change. In this case, the stationary distribution of linking dynamics determines the average fitness of individuals [39]. Then, we can employ the strategy dynamics from well mixed populations for our structured system. The average payoff of cooperators is given by
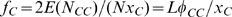
(13)The average payoff of defectors is
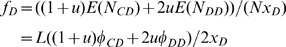
(14)


Equating 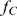 and 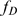 or, equivalently, substituting them into Eq. (12), we find that an unstable equilibrium 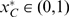 emerges when
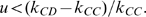
(15)It is located at
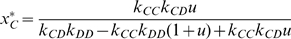
(16)This critical value 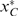 determines the attraction basin of cooperation 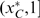: Cooperators take over when their initial frequency 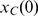 is larger than this critical value, whereas defectors take over when 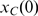 is less than this critical value. In other words, the evolutionary PD game with linking dynamics is similar to that of the coordination game in well mixed population where both cooperation and defection are best replies to themselves.

Let us show how the PD game transforms into a coordination game under linking dynamics. Substituting Eqs. (13)(14) into Eq. (12) yields
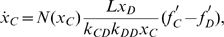
(17)where the first factors are always positive and 
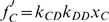
 and 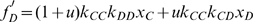 are the payoffs of cooperators and defectors in a modified game with payoff matrix
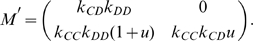
(18)In other words, the coevolution of strategy and structure transforms the original PD game into another one. In particular, 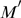 turns to a coordination game when 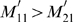, i.e., 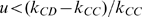. Thus, the PD game with linking dynamics corresponds to a coordination game in a well mixed population [39], [40]. Cooperation is stable only when
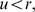
(19)where 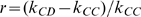.

The quantity 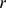 measures the propensity for cooperators to form clusters that supports cooperation [16]. Indeed, remembering that 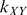 indicates the probability with which an 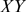 link breaks, 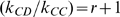 characterizes the fragility ratio between 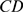 and 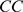 link. In particular, 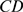 link are more fragile than 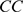 links if 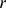 exceeds zero. In other words, a cooperator is more likely to play with cooperators than defectors and to sustain the social relationship when 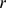 is greater than zero. Therefore 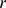 also illustrates how likely a cooperator is to interact with a cooperator. The greater 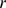, the more likely it is for cooperators to form clusters.

Increasing 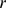 allows cooperators to spread more effectively and can allow them to invade from initially small clusters [52]. The quantity 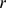 characterizes the propensity of cooperators to form clusters. Cooperation gains a foothold when 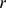 is sufficiently large. Precisely, 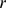 is sufficiently large when 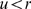 by Eq. (19). In this case, cooperator clusters expand and take over the whole population.

We have explained intuitively how 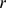 enhances cooperation level. In addition, we can also show analytically that large 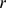 leads to cooperation by enlarging the cooperation attraction basin:

Substituting 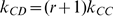 to Eq. (16), we obtain:
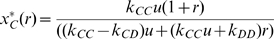
(20)The quantity 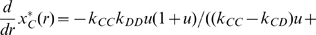 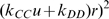 is always negative for all permitted parameters. Hence, 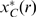 is a decreasing function of 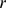. Since 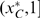 is the attraction basin of cooperation. Accordingly, increasing 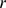 enlarges the attraction basin of cooperation. In other words, it requires fewer cooperators to take over the whole population with larger 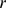.

**Figure 2:**
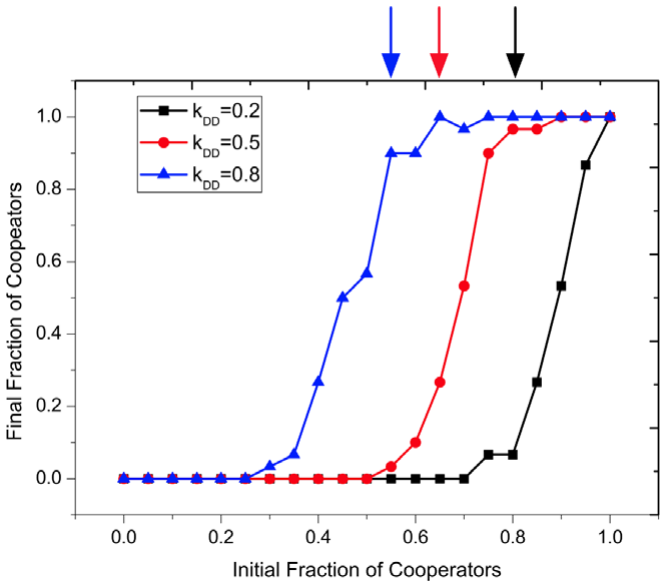
Final fraction of cooperators as a function of initial fraction of cooperators. The symbols indicate the simulation while the arrows represent the analytical results. Both simulation and the analytical results show that fewer cooperators can invade a population of defectors when the 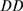 ties are more fragile, which validates the analytical prediction. 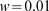, 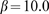, 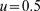, 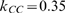, 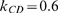, 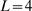 and 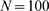 for all the three lines in the plot. In addition, each data point for all the plots from Fig. (2) to [Fig pone-0011187-g003] [Fig pone-0011187-g004] [Fig pone-0011187-g005] Fig. (6) is averaged over 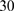 independent runs. And in each run, we set the mean value over time window of 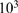 generations to be the final fraction of cooperators, after a transient time of 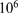 generations.

So far, it has been shown that the simple rule gives us an insight on how cooperation comes into being with linking dynamics. Furthermore, it can also be revealed that 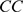 links should be less fragile [39] while 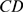 ones should be easy to break in order to promote cooperation. Since 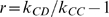, the larger 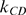 or the smaller 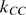, the greater 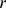 is. Thus cooperation is promoted when the probability to break 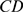 links 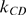 is large or the probability to break 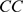 links 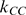 is small. This is in line with previous numerical consideration [37], [38]. However, 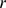 is independent of 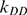. Does this mean that 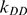 has no impact on cooperation? In fact, it is not the case. On the contrary, 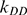 plays an important role in promoting cooperation when 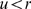 holds. Actually, this simple rule only guarantees that the equilibrium 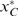 of Eq. (16) lies between zero and one, where it is defined. However it is not sufficient to make cooperation advantageous. Besides, the initial frequency of cooperators should lie in the attraction basin of cooperation 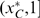 to make cooperators gain a foothold in the population. Nevertheless, notice that Eq. (16) can be rewritten as:
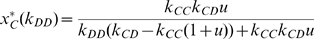
(21)hence, 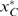 is a decreasing function of 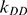 provided 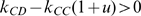, i.e. the simple rule holds. In this way, increasing 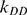 augments the attraction basin of cooperation 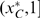 (See Fig. (2)). Thus it is easier for cooperators to gain a foothold when 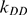 is larger.

**Figure 3 pone-0011187-g003:**
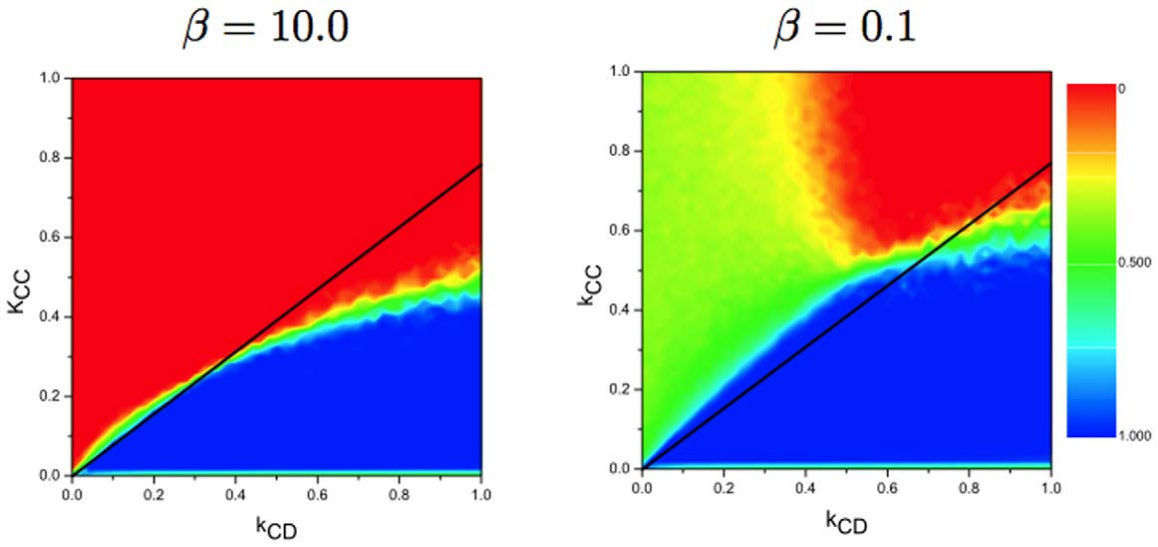
Results for the final fraction of cooperators for different selection pressure 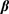. It shows how the selection pressure affects the analytical prediction. The black line is our analytical condition 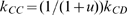. Initially, all the individuals are situated on a regular graph of degree 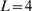 and size 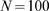. Each individual is assigned to be a cooperator or a defector with the same probability. All plots from Fig. (3) to [Fig pone-0011187-g004] [Fig pone-0011187-g005] Fig. (6) share the same color code and initial condition. Analytical results predict that higher cooperation level can emerge only below the black line. Simulation results show that the analytical result is more accurate for strong selection than weak selection as expected. The error is induced by the finite population size effect. (other parameters 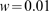, 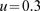, and 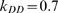).

In Fig. (3), we show that the simulation results are in agreement with our analytical predictions when the selection pressure is high, while the simulations deviate from the analytical results when the selection pressure is low. For strong selection, we find above the line 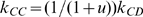, the cooperation level is low, which is consistent with our theoretical predictions. For weak selection, however, the cooperation level is almost 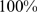 for the parameter region closely above the line for 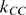 between 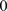 and 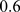, where the cooperation level should be low based on our the simple rule.

These deviations are due to both the finite population effect and the approximation of linking dynamics by Eq. (5). On the one hand, as mentioned above, the transition matrix Eq. (6) is only an approximation based on the global frequency of cooperators, while they are also influenced by local frequencies in the simulations. On the other hand, we use the replicator equation to describe the strategy evolution. But the replicator equation is only an approximation of the strategy evolution when the population size is sufficiently large, which implies that small fitness differences can influence the dynamics. This explains why our theoretical predictions are less accurate for weak selection. Therefore, we focus on strong selection in the following.

**Figure 4 pone-0011187-g004:**
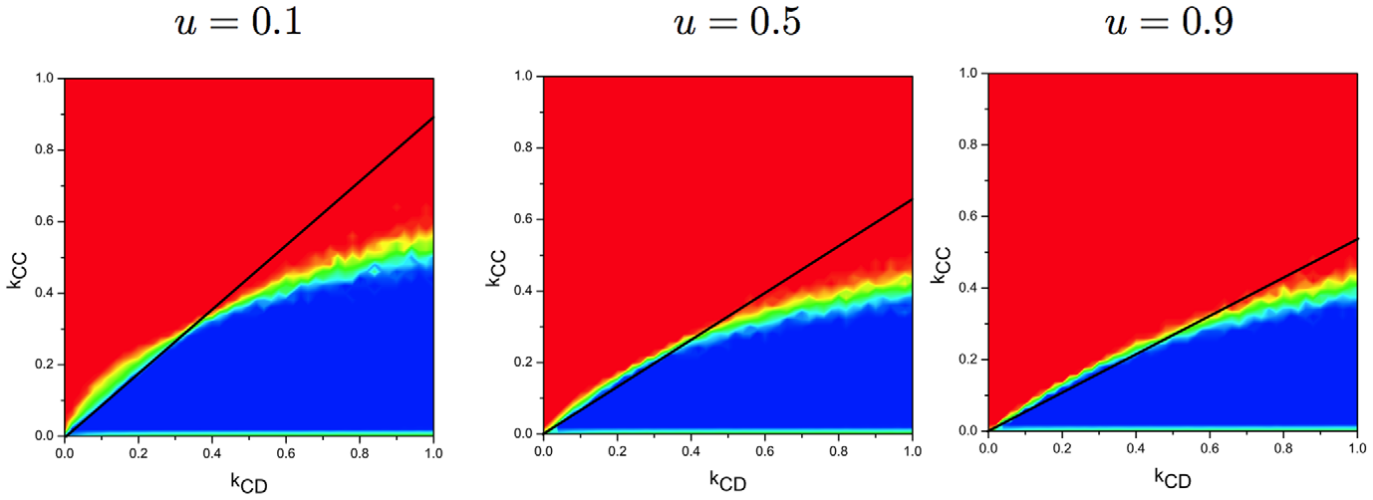
Results for the final fraction of cooperators for different values of the payoff parameter 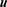. The slope of the critical line is increasing when 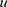 is decreasing, indicating that cooperators are more likely to emerge when the parameter 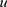 is small. (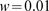, 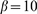 and 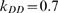. )

We first investigate how 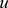 affects the evolution of cooperation. For each plot in Fig. (4), above the line 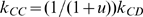, there is nearly no cooperation, while below the line, cooperation is possible. This is consistent with our simple rule. Furthermore, compared with the three plots in Fig. (4), we observe a decrease of the parameter region to sustain the cooperation when 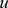 increases. It indicates that only a small temptation to defect can sustain cooperation.

**Figure 5 pone-0011187-g005:**
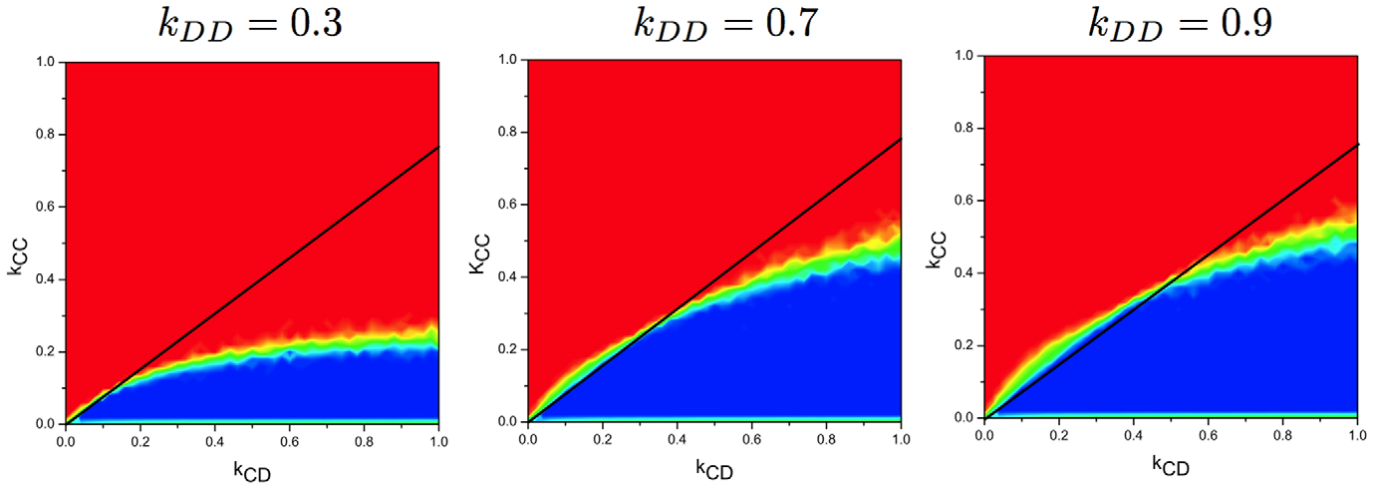
Results for the final fraction of cooperators for different values of 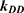. It shows that quick partner switching between defectors, i.e., high 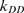, promotes cooperation. (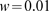, 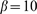 and 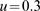.)

Let us further examine the role of 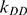 in the evolution of cooperation by simulation. It is observed clearly in Fig. (5) that the more fragile 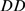 ties are, the easier it is for cooperators to wipe out defectors. Intuitively, for greater 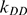, 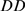 links are more likely to break and defectors are no longer trapped in their fruitless interactions and can instead seek new cooperators to exploit. Thus, it seems less likely to promote cooperation for large 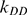. However, both analytical and simulation results show that high 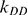 promotes cooperation (See Eq. (21) and Fig. (2)). This is counter-intuitive. In fact, in this case, the quick partner-switching between defectors induces the heterogeneity of the population, which results in cooperation. Similar results have been reported in [43].

**Figure 6:**
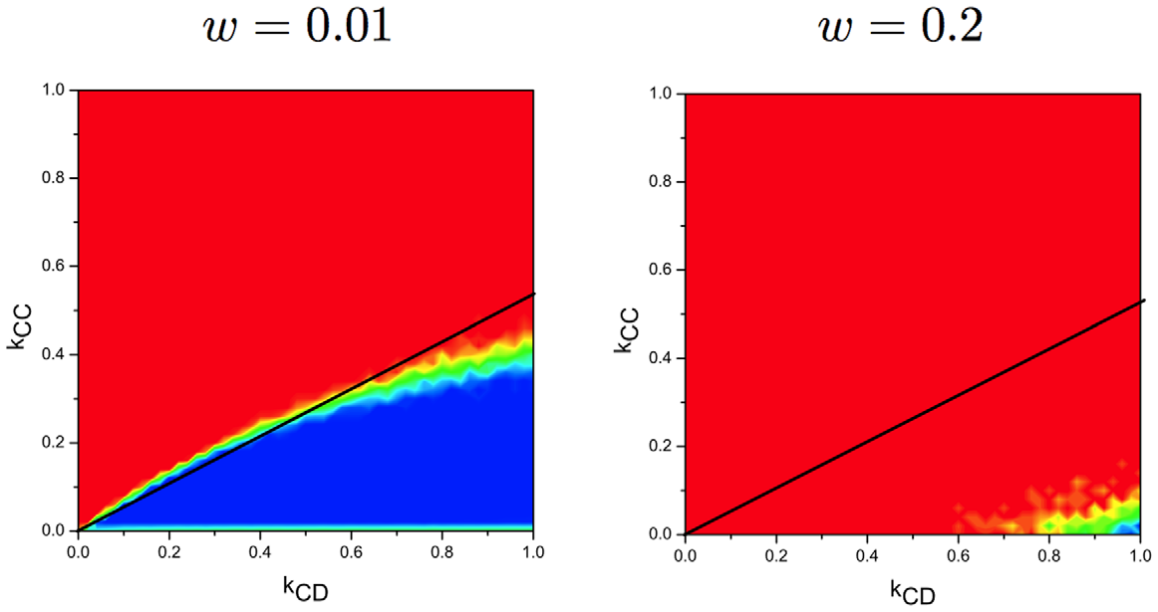
Results for the final fraction of cooperators for different values of 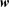. Our analytical results are only valid under time scale separation, as shown in this plot. (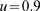, 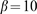 and 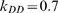)

Finally, we turn to investigate the role of 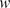 on the coevolution. Fig. (6) shows that for small 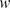, the result is in good agreement with the theoretical prediction, while deviates from the simple rule for large 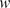, as expected. Similar results have been reported in the analytical approach of Pacheco et al. [39]. Both analytical approaches are based on the time scale separation, i.e., all the links are almost in the stationary states when the strategy update occurs.

## Results and Discussion

To sum up, we have established a discrete model to describe the stochastic linking dynamics analytically in terms of a Markov chain. Based on this linking dynamics, we have studied the coevolution of strategy and network structure. A simple condition for the evolution of cooperation is obtained analytically that becomes more accurate when selection is stronger. The rule shows that the less fragile 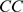 links are, the easier cooperation emerges. The more fragile 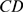 links are, the easier cooperation prevails.

Compared to Pacheco *et al.*'s work, time scales separation also plays an important part in our analysis. In Pacheco *et al.*'s work, time separation is used to ensure that the linking dynamics is in the stationary regime when the strategy evolution happens. But in contrast to Pacheco *et al.*'s work, our analytical approach explicitly considers stochastic effects in the linking dynamics. Further, when the population size is sufficiently large, this Markov chain describing the linking dynamics can be approximated by a different system of differential equations. Since the total number of links is constant in our approach, there are only two independent variables describing the different kinds of links. In Pacheco *et al.*'s method, however, all the three variables are independent. In general, both methods lead to very similar qualitative results.

Regarding the coevolution of strategy and network, previous numerical work with constant number of links has assumed that dissatisfied ties are more likely to break off than satisfied ones. In this case, cooperation is more likely to be sustained. However, it has not been shown analytically that to what extent satisfied links are more stable than adverse ones to make cooperation gain a foothold. The simple rule 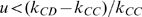 reveals such a relation between the payoff matrix and the parameters of the linking dynamics. It shows under which conditions cooperation may prevail, provided the linking dynamics is sufficiently fast. Furthermore, we have provided a series of numerical results to validate the analytical results. We find that numerical results are in agreement with the analytical results for strong selection, yet may deviate from the analytical results for weak selection.
